# Single-crystalline nanoporous Nb_2_O_5 _nanotubes

**DOI:** 10.1186/1556-276X-6-138

**Published:** 2011-02-14

**Authors:** Jun Liu, Dongfeng Xue, Keyan Li

**Affiliations:** 1 State Key Laboratory of Fine Chemicals, Department of Materials Science and Chemical Engineering, School of Chemical Engineering, Dalian University of Technology, Dalian 116024, People's Republic of China

## Abstract

Single-crystalline nanoporous Nb_2_O_5 _nanotubes were fabricated by a two-step solution route, the growth of uniform single-crystalline Nb_2_O_5 _nanorods and the following ion-assisted selective dissolution along the [001] direction. Nb_2_O_5 _tubular structure was created by preferentially etching (001) crystallographic planes, which has a nearly homogeneous diameter and length. Dense nanopores with the diameters of several nanometers were created on the shell of Nb_2_O_5 _tubular structures, which can also retain the crystallographic orientation of Nb_2_O_5 _precursor nanorods. The present chemical etching strategy is versatile and can be extended to different-sized nanorod precursors. Furthermore, these as-obtained nanorod precursors and nanotube products can also be used as template for the fabrication of 1 D nanostructured niobates, such as LiNbO_3_, NaNbO_3_, and KNbO_3_.

## Introduction

Nanomaterials, which have received a wide recognition for their size- and shape-dependent properties, as well as their practical applications that might complement their bulk counterparts, have been extensively investigated since last century [1-8]. Among them, one-dimensional (1D) tubular nanostructures with hollow interiors have attracted tremendous research interest since the discovery of carbon nanotubes [1,9-14]. Most of the available single-crystalline nanotubes structurally possess layered architectures; the nanotubes with a non-layered structure have been mostly fabricated by employing porous membrane films, such as porous anodized alumina as template, which are either amorphous, polycrystalline, or only in ultrahigh vacuum [13,14]. The fabrication of single-crystalline semiconductor nanotubes is advantageous in many potential nanoscale electronics, optoelectronics, and biochemical-sensing applications [1]. Particularly, microscopically endowing these single-crystalline nanotubes with a nanoporous feature can further broaden their practical applications in catalysis, bioengineering, environments protection, sensors, and related areas due to their intrinsic pores and the high surface-to-volume ratio. However, it still remains a big long-term challenge to develop those simple and low-cost synthetic technologies to particularly fabricate 1 D nanotubes for functional elements of future devices. Recently, the authors have rationally designed a general thermal oxidation strategy to synthesize polycrystalline porous metal oxide hollow architectures including 1 D nanotubes [15]. In this article, a solution-etching route for the fabrication of single-crystalline nanoporous Nb_2_O_5 _nanotubes with NH_4_F as an etching reagent, which can be easily transformed from Nb_2_O_5 _nanorod precursors is presented.

As a typical *n*-type wide bandgap semiconductor (*E*_g _= 3.4 eV), Nb_2_O_5 _is the most thermodynamically stable phase among various niobium oxides [16]. Nb_2_O_5 _has attracted great research interest due to its remarkable applications in gas sensors, catalysis, optical devices, and Li-ion batteries [9-11,16-21]. Even monoclinic Nb_2_O_5 _nanotube arrays were successfully synthesized through a phase transformation strategy accompanied by the void formation [10], which can only exist as non-porous polycrystalline nanotubes. In this study, a new chemical etching route for the synthesis of single-crystalline nanoporous Nb_2_O_5 _nanotubes, according to the preferential growth habit along [001] of Nb_2_O_5 _nanorods, is reported. The current chemical etching route can be applied to the fabrication of porous and tubular features in single-crystalline phase oxide materials.

## Experimental section

### Materials synthesis

#### Nb_2_O_5 _nanorod precursors

Nb_2_O_5 _nanorods were prepared via hydrothermal technique in a Teflon-lined stainless steel autoclave. In a typical synthesis of 1 D Nb_2_O_5 _nanorods, freshly prepared niobic acid (the detailed synthesis processes of niobic acid from Nb_2_O_5 _has been described in previous studies by the authors [22-25]) was added to the mixture of ethanol/deionized water. Subsequently, the white suspension was filled into a Teflon-lined stainless steel autoclave. The autoclave was maintained at 120-200°C for 12-24 h without shaking or stirring during the heating period and then naturally cooled down to room temperature. A white precipitate was collected and then washed with deionized water and ethanol. The nanorod precursors were dried at 60°C in air.

#### Single-crystalline nanoporous Nb_2_O_5 _nanotubes

In a typical transformation, 0.06-0.20 g of the obtained Nb_2_O_5 _nanorods was added to 20-40 ml deionized water at room temperature. 2-8 mmol NH_4_F was then added while stirring. Afterward, the mixture was transferred into a Teflon-lined stainless steel autoclave and kept inside an electric oven at 120-180°C for 12-24 h. Finally, the resulting Nb_2_O_5 _nanotubes were collected, and washed with deionized water and ethanol, and finally dried at 60°C in air.

## Materials characterization

The collected products were characterized by an X-ray diffraction (XRD) on a Rigaku-DMax 2400 diffractometer equipped with the graphite monochromatized Cu Kα radiation flux at a scanning rate of 0.02°s^-1^. Scanning electron microscopy (SEM) analysis was carried using a JEOL-5600LV scanning electron microscope. Energy-dispersive X-ray spectroscopy (EDS) microanalysis of the samples was performed during SEM measurements. The structures of these nanorod precursors and nanotube products were investigated by means of transmission electron microscopy (TEM, Philips, TecnaiG2 20). UV-Vis adsorption spectra were recorded on UV-Vis-NIR spectrophotometer (JASCO, V-570). The photoluminescence (PL) spectrum was measured at room temperature using a Xe lamp with a wavelength of 325 nm as the excitation source.

## Results and discussion

**Figure 1:**
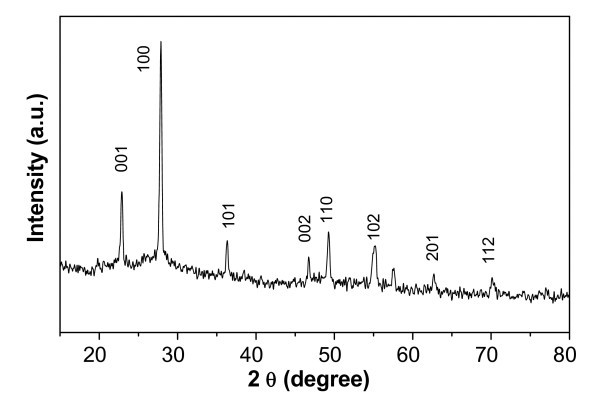
**XRD pattern of Nb_2_O_5 _nanorod precursors**. All the peaks can be indexed to the orthorhombic Nb_2_O_5 _(JCPDS no. 30-0873).

**Figure 2:**
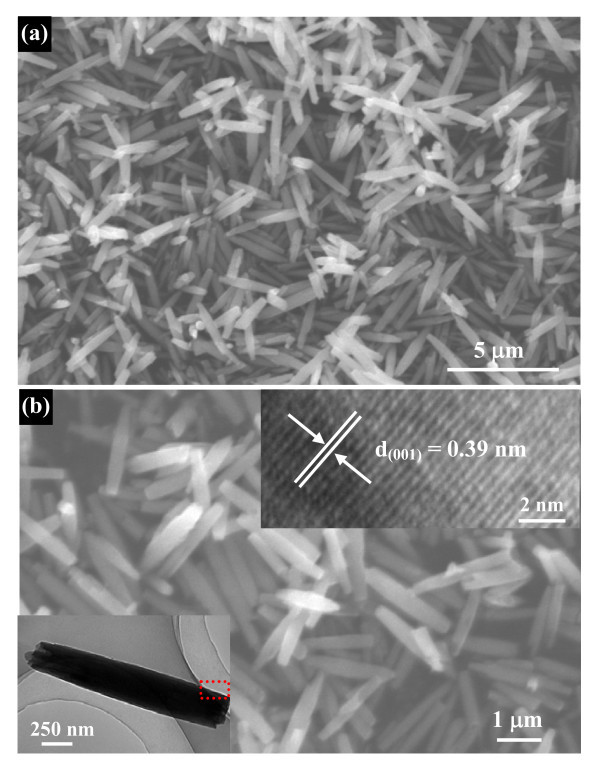
**Morphology and structure characterizations of Nb_2_O_5 _nanorod precursors**: **(a) **low-magnification SEM image shows that these precursor nanorods have a uniform diameter and length; **(b) **high-magnification SEM image. The bottom inset is a low-magnification TEM image of a single solid nanorod. The top inset shows a HRTEM image of the boxed region shown in the bottom inset of Figure 2c, which indicates that these precursor nanorods grow along the [001] direction.

Typical XRD pattern of the Nb_2_O_5 _nanorod precursors obtained from the ethanol-water system shown in Figure 1 exhibits diffraction peaks corresponding to the orthorhombic Nb_2_O_5 _with lattice constants of *a *= 3.607 Å and *c *= 3.925 Å (JCPDS no. 30-0873). No diffraction peaks arising from impurities such as NbO_2 _were detected, indicating the high purity of these precursor nanorods. The morphology of these precursor products was observed by means of SEM and TEM. Figure 2 shows typical SEM images of the obtained Nb_2_O_5 _precursors with uniform 1 D rod-like morphology. The high magnification image (Figure 2b) clearly displays these nanorods with the diameter 300-600 nm and the length 2-4 μm. The bottom inset of Figure 2b shows typical TEM image of a single solid Nb_2_O_5 _nanorod, demonstrating that the nanorod have a diameter of ~300 nm and length of approximately 2 μm, which is in agreement with the SEM observations. The HRTEM image (the top inset of Figure 2b) taken from the square area exhibits clear lattice fringes, indicating that the nanorod is highly crystallized. The spacing of 0.39 nm corresponds to the (001) planes of Nb_2_O_5_, which shows that these precursor nanorods grow along the [001] direction.

**Figure 3:**
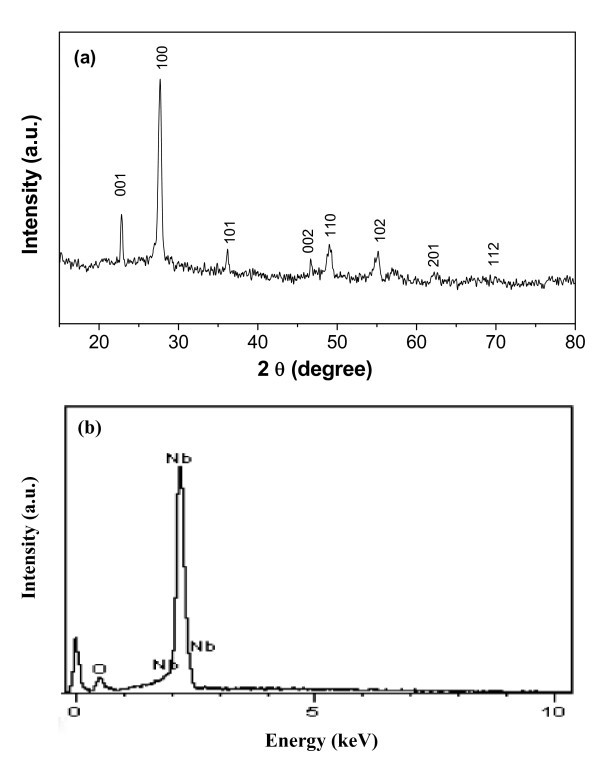
**Composition characterizations of Nb_2_O_5 _nanotube products: XRD (**a**) and EDX (**b**) patterns of single-crystalline nanoporous Nb_2_O_5 _nanotubes**. All the peaks in Figure 3a totally overlap with those of pure Nb_2_O_5 _(compare reference lines, JCPDS no. 30-0873) and no evidence of any impurity was detected.

After the hydrothermal process along with an interface reaction, Nb_2_O_5 _nanotubes were obtained with F^-^-assisted etching treatment. The XRD pattern shown in Figure 3a reveals a pure phase, and all the diffraction peaks are very consist with that of nanorod precursors and the reported XRD profile of the orthorhombic Nb_2_O_5 _(JCPDS no. 30-0873). EDS analysis was used to determine the chemical composition of an individual nanotube. The result shows that these nanotube products contain only Nb and O elements, and their atomic ratio is about 2:5, which is in agreement with the stoichiometric ratio of Nb_2_O_5_. The EDS results clearly confirm that F was not doped into these nanotubes (Figure 3b).

**Figure 4:**
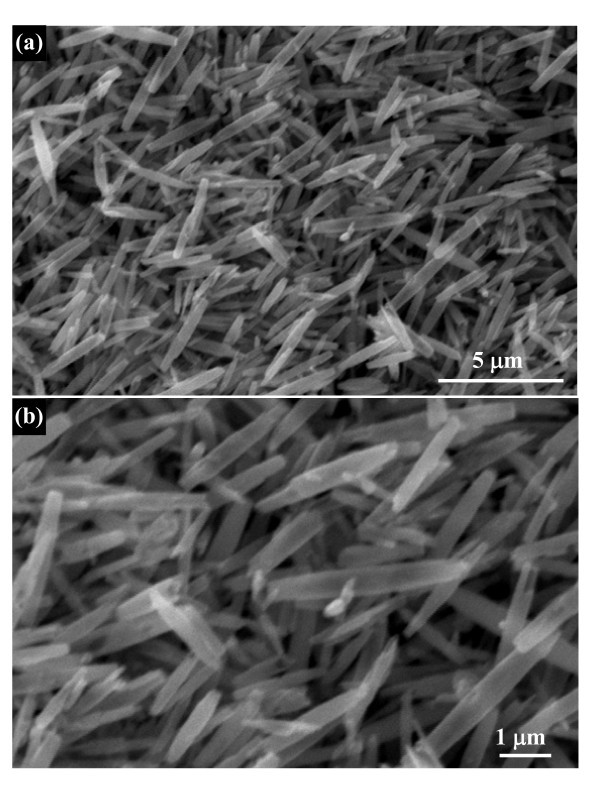
**SEM images of single-crystalline nanoporous Nb_2_O_5 _nanotubes**: **(a) **low-magnification SEM image; **(b) **high-magnification SEM image.

**Figure 5:**
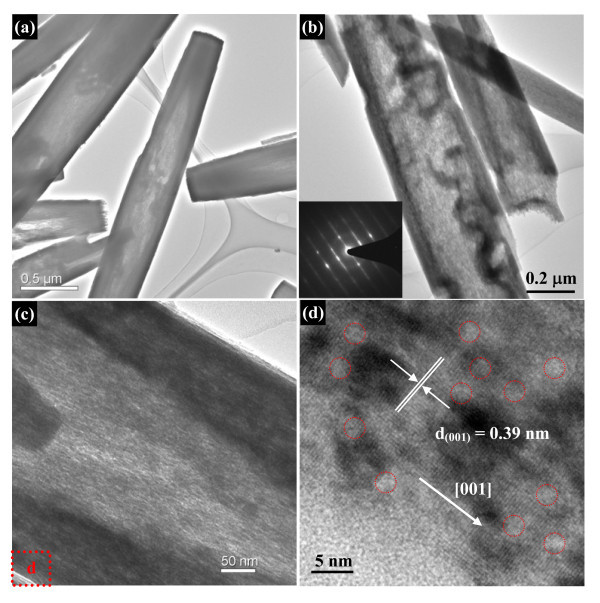
**TEM characterizations of single-crystalline nanoporous Nb_2_O_5 _nanotubes**: **(a) **low-magnification TEM image of nanoporous Nb_2_O_5 _nanotubes; **(b, c) **high-magnification TEM images of nanoporous Nb_2_O_5 _nanotubes showing that these nanotubes have a nanoporous shell. The inset of Figure 5b shows the SAED pattern taken from an individual nanotube indicating that these nanotubes are single-crystalline; **(d) **HRTEM image of the porous shell of a single nanotube revealing (001) lattice planes. The red circles indicate that the shell of these nanotubes densely distributes nanopores.

**Figure 6:**
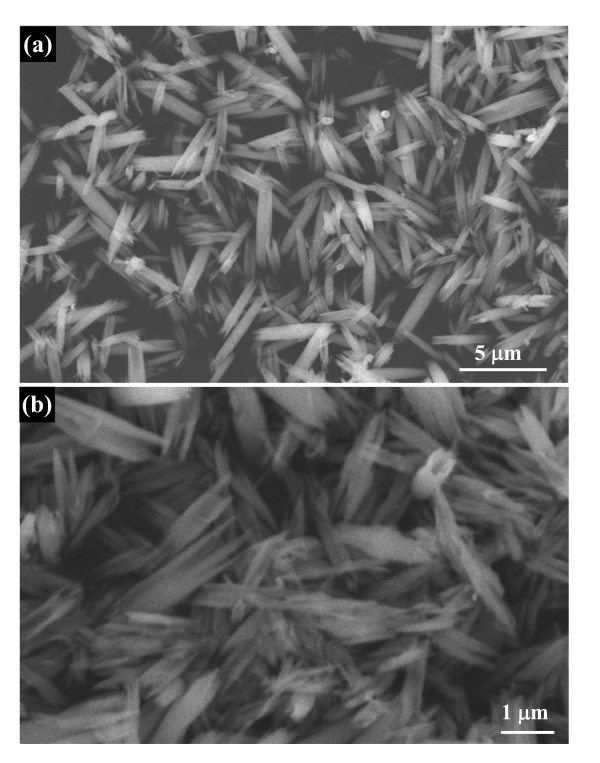
**SEM images of collapsed Nb_2_O_5 _nanotubes obtained with HF as etching reagent**: **(a) **low-magnification SEM image; **(b) **high-magnification SEM image.

**Figure 7:**
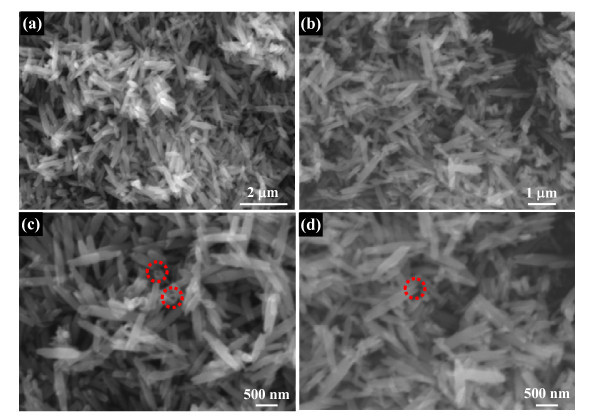
**SEM images of Nb_2_O_5 _nanotubes with a smaller diameter (approximately 200 nm)**. These nanotubes products were obtained with the same etching route. Red circles in Figure 7c and d indicate the hollow section of nanotubes.

The morphology and structure of the finally nanoporous nanotubes were first evaluated by SEM observation. The representative SEM image in Figure 4a reveals the presence of abundant 1 D rod-like nanostructure, implying the finally formed nanotubes well resemble the shape and size of Nb_2_O_5 _nanorod precursors. The detailed structure information is supported by the high-magnification image shown in Figure 4b, which shows some typical nanotubes with thin walls. For accurately revealing the microstructure of these nanotubes, TEM observation was performed on these nanotubes. Figure 5a shows a typical TEM image of these special nanostructured Nb_2_O_5_. These nanotubes have a hollow cavity and two closed tips. A magnified TEM image of some Nb_2_O_5 _nanotubes is presented in Figure 5b. It can been see that the nanotube surface is highly nanoporous and coarse, composed of dense nanopores. SAED pattern obtained from them by TEM shows they are single-crystalline, as seen in the typical pattern in Figure 5b (inset). The nanoporous characterization of these single-crystalline nanotubes was further verified by a higher-magnified TEM image (Figure 5c). The single-crystalline nature of the nanotubes is further indicated by the Nb_2_O_5 _lattice which can be clearly seen in the HRTEM image of the surface of a nanoporous nanotube. Though it is difficult to directly observe by TEM, since the observed image is a two-dimensional projection of the nanotubes, Figure 5d shows dense nanopores around which the Nb_2_O_5 _lattice is continuous. The diameter of the nanopores appears to be 2-4 nm, and the growth direction of these nanoporous nanotubes is [001], just the same as nanorod precursors. During the hydrothermal process of Nb_2_O_5 _nanorod precursors, the formation of single-crystalline nanoporous nanotubes can be ascribed to preferential-etching of single-crystalline nanorods. In hydrothermal aqueous NH_4_F solution, HF were formed by the hydrolysis of NH_4_^+ ^and were further reacted with Nb_2_O_5 _to form soluble niobic acid. The etching of nanorods in this study preferentially begins at the central site of the nanorod, which might be because the central site has high activity or defects both for growth and for etching. Further etching at the center of nanorod leads to its splitting, and the atom in the (001) planes are removed at the next process, causing the formation of the tubular structure. Furthermore, during the etching process, these newly generated soluble niobic acid diffused into the reaction solution from the central of the precursor nanorods, leaving dense nanopores on the shell of nanotubes with closed tips. For verifying such preferential-etching formation mechanism, HF solution as an etching reagent was directly adopted. Figure 6 shows the morphology and structure of Nb_2_O_5 _products, which exhibit that hollow tuber-like nanostructures can also be achieved. However, the as-obtained Nb_2_O_5 _products are broken or collapsed nanotubes, which is ascribed to the fast etching rate of HF reagent. The diameter of nanoporous nanotubes can be tunable by adjusting the diameter of precursor nanorods. We can thus obtain different diameters of Nb_2_O_5 _nanotubes, which could meet various demands of nanotubes toward practical applications. For example, when Nb_2_O_5 _nanorods with a smaller diameter (approximately 200 nm) were adopted as precursors, the corresponding Nb_2_O_5 _nanotubes with similar sized nanotubes were achieved (Figure 7).

**Figure 8:**
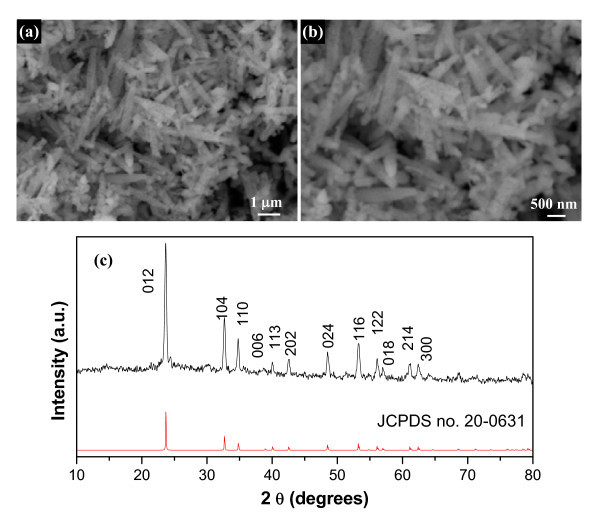
**Morphology and composition characterizations of LiNbO_3 _nanorods**. SEM images **(a, b) **and XRD pattern **(c) **of LiNbO_3 _nanorods obtained through calcination of Nb_2_O_5 _nanorod precursors and LiOH at 500°C for 4 h. All the peaks in Figure 8c totally overlap with those of the rhombohedral LiNbO_3 _(JCPDS no. 20-0631), and no evidence of impurities was detected.

These Nb_2_O_5 _nanotubes and nanorods can be used as versatile templates to fabricate MNbO_3 _(M = Li, Na, K) nanotubes and nanorods. For example, when Nb_2_O_5 _nanorod precursors directly reacted with LiOH at high temperature, LiNbO_3 _nanorods were immediately achieved. As shown in Figure 8a, b, the morphology of Nb_2_O_5 _templates is preserved. XRD pattern of the calcination products (Figure 8c) clearly shows the pure-phase LiNbO_3 _ferroelectric materials. These LiNbO_3 _nanorods were obtained through calcination of Nb_2_O_5 _and LiOH with appropriate amount ratios at 500°C for 4 h. This calcination method is general and versatile, and it can be applied to fabricate other niobate materials such as NaNbO_3 _and KNbO_3_. The optical properties of these Nb-based nanomaterials (LiNbO_3_, NaNbO_3_, and KNbO_3_) are shown in Figure S1 in Additional file 1).

**Figure 9:**
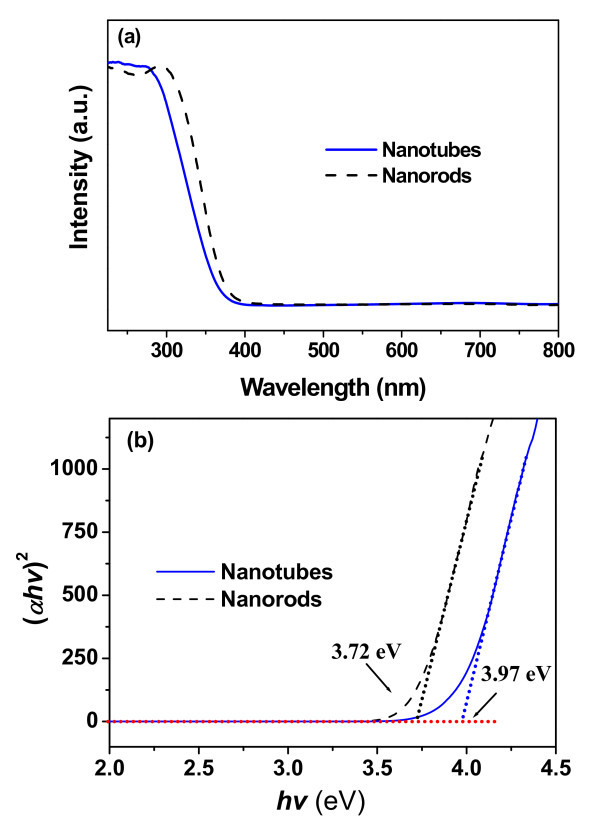
**Optical properties of Nb_2_O_5 _nanorod precursors and nanotube products**. UV-Vis spectra (**a**) and the corresponding (αhv)^2 ^versus photo energy (*hv*) plots (**b**) of Nb_2_O_5 _nanorods and nanotubes measured at room temperature.

UV-Vis adsorption measurement was used to reveal the energy structure and optical property of the as-prepared Nb_2_O_5 _nanorods and finally porous nanotube products. UV-Vis adsorption spectra of Nb_2_O_5 _nanorods and nanotubes are presented in Figure 9a. It can be seen from Figure 9a that the structure transformation from solid nanorods to nanoporous nanotubes is accompanied by distinct changes in the UV-Vis spectra because of the significant difference in shape between nanorod precursors and nanotube products. As a direct band gap semiconductor, the optical absorption near the band edge follows the formula

(1) $$\alpha hv=A{\left(hv-E_{\text{g}}\right)}^{\text{1}/\text{2}}$$

where α, *v*, *E*_g_, and *A *are the absorption coefficient, light frequency, band gap energy, and a constant, respectively [16,26]. The band gap energy (*E*_g_) of Nb_2_O_5 _can be defined by extrapolating the rising part of the plots to the photon energy axis. The estimated band gaps of Nb_2_O_5 _nanotubes and nanorods are 3.97 and 3.72 eV, respectively (Figure 9b), which are both larger than the reported value (3.40 eV) of bulk crystals [10]. The blue shift (approximately 0.25 eV) of the absorption edge for the porous nanotubes compared to solid nanorods exhibits a possible quantum size effect in the orthorhombic nanoporous Nb_2_O_5 _nanotubes [10]. Wavelength and intensity of absorption spectra of Nb_2_O_5 _nanocrystals depend on the size, crystalline type and morphology of the Nb_2_O_5 _nanocrystals. If their size is smaller, then the absorption spectrum of Nb_2_O_5 _nanocrystals becomes blue shifted. The spectral changes are observed because of the formation of nanoporous thin-walled tubular nanomaterials, similar to the previous research result [10].

## Conclusions

In summary, we have elucidated a new preferential-etching synthesis for single-crystalline nanoporous Nb_2_O_5 _nanotubes. The shell of resulting nanotubes possesses dense nanopores with size of several nanometers. The formation mechanism of single-crystalline nanoporous nanotubes is mainly due to the preferential etching along *c*-axis and slow etching along the radial directions. The as-obtained Nb_2_O_5 _nanorod precursors and nanotube products can be used as templates for synthesis of 1 D niobate nanostructures. These single-crystalline nanoporous Nb_2_O_5 _nanotubes might find applications in catalysis, nanoscale electronics, optoelectronics, and biochemical-sensing devices.

## Abbreviations

EDS: Energy-dispersive X-ray spectroscopy; PL: photoluminescence; 1D: one-dimensional; SEM: Scanning electron microscopy.

## Competing interests

The authors declare that they have no competing interests.

## Authors' contributions

JL carried out the sample preparation. JL and KL participated in the UV-Vis and PL measurements. JL carried out the XRD, SEM, TEM and EDS mesurements, the statistical analysis and drafted the manuscript. DX conceived of the study and participated in its design and coordination. All authors read and approved the final manuscript.

## Supplementary Material

Additional file 1 **Figure S1 UV-Vis (a) and PL (b) spectra of Nb-based nanomaterials**. PL spectra were obtained with an excitation wavelength of 325 nm measured at room temperature. Click here for file
