# Supplementary figures and images for: Single-crystalline nanoporous Nb2O5 nanotubes
Source: Nanoscale Res Lett. 2011 Feb 14;6(1):138. doi: 10.1186/1556-276X-6-138 (PMC3211185; doi:10.1186/1556-276X-6-138)

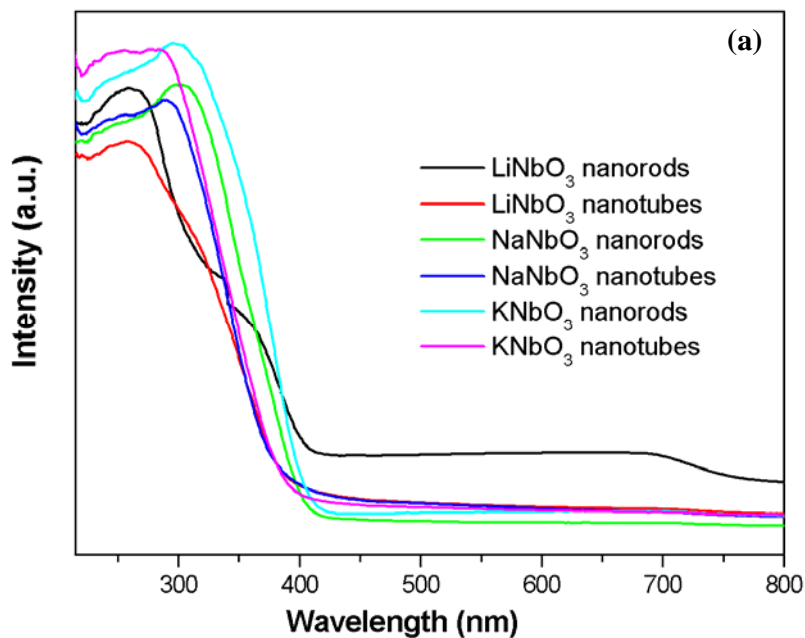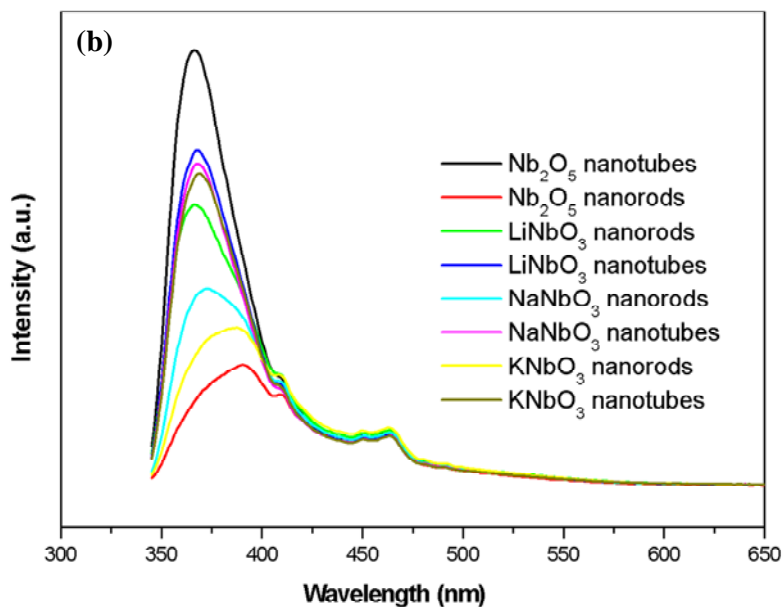

Supplement: Additional file 1 — Figure S1 UV-Vis (a) and PL (b) spectra of Nb-based nanomaterials. PL spectra were obtained with an excitation wavelength of 325 nm measured at room temperature. [file 1556-276X-6-138-S1.PDF]
